# Non-Invasive Approaches for the Evaluation of the Functionalization of Melamine Foams with In-Situ Synthesized Silver Nanoparticles

**DOI:** 10.3390/polym12050996

**Published:** 2020-04-25

**Authors:** Suset Barroso-Solares, Paula Cimavilla-Roman, Miguel Angel Rodriguez-Perez, Javier Pinto

**Affiliations:** 1 Cellular Materials Laboratory (CellMat), Condensed Matter Physics Department, University of Valladolid, 47011 Valladolid, Spain; 2 BioecoUVA Research Institute, University of Valladolid, 47011 Valladolid, Spain; 3 Group UVASENS, Escuela de Ingenierías Industriales, University of Valladolid, Paseo del Cauce, 59, 47011 Valladolid, Spain

**Keywords:** noble metal nanoparticles, polymer foam, nanocomposites, colorimetry, X-ray radiography

## Abstract

The use of polymeric nanocomposites has arisen as a promising solution to take advantage of the properties of nanoparticles (NPs) in diverse applications (e.g., water treatment, catalysis), while overcoming the drawbacks of free-standing nanoparticles (e.g., aggregation or accidental release). In most of the cases, the amount and size of the NPs will affect the stability of the composite as well as their performance. Therefore, a detailed characterization of the NPs present on the nanocomposites, including their quantification, is of vital importance for the optimization of these systems. However, the determination of the NPs load is often carried out by destructive techniques such as TGA or ICP-OES, the development of non-invasive approaches to that aim being necessary. In this work, the amount of silver NPs synthesized directly on the surface of melamine (ME) foams is studied using two non-invasive approaches: colorimetry and X-ray radiography. The obtained results show that the amount of silver NPs can be successfully determined from the luminosity and global color changes of the surface of the foams, as well as from the X-ray attenuance.

## 1. Introduction

In the past decades, the use of porous polymer structures as host materials for the production of nanocomposites has become a relevant field of research [1,2,3,4,5,6,7,8]. Among the most effective approaches to fabricate porous polymer nanocomposites, the following can be mentioned: mechanical blending [2,7,9] and surface functionalization processes [8,10] such as dip-coating [11,12], spray-coating [5], or in situ synthesis [3,6,10]. These procedures aim to take advantage of the features of both matrix and filler of the nanocomposites. On the one hand, the porous polymer provides a large surface area and excellent mechanical strength for long-term use. On the other hand, the anchoring of the nanoparticles (NPs) to the matrix avoids risks of their accidental release to the environment [4,13,14], as well as their efficiency loss in some applications (e.g., water treatment, catalysis) due to aggregation [6,15,16].

However, a critical factor in keeping the functionality of the filler is a proper selection of the nanocomposite fabrication route. First, the choice of the porous polymeric matrix is strongly linked to the application. The wide availability of diverse polymers allows providing matrices with optimal features for each application, in terms of the porous structure, mechanical and thermal behavior, wettability, chemical stability, or even bio-compatibility or bio-degradability [1,4,17,18]. Second, the method selected to incorporate the filler has a crucial effect on the final properties of the nanocomposite, as most of their potential applications (e.g., water treatment, catalysis, sensoring) could require the presence of the filler on the external surface of the polymeric substrate [2,10]. Finally, it is fundamental to select a filler able to provide the desired functionality and compatible with both the polymer matrix and production route. A broad range of nanofillers can be found in the literature, including clays, [2,18,19,20] organic compounds, [11] graphene, [5,8,12], and inorganic nanoparticles (e.g., metal nanoparticles) [1,4,6,21]. In particular, noble metal nanoparticles are of great interest for their remarkable physical properties [16] such as their stability, large surface area, surface-enhanced Raman scattering, [21] surface plasmon resonance [15,21], and high interfacial reactivity [16,22,23,24,25]. Accordingly, these nanoparticles can be employed in diverse applications such as water treatment [1,6,24,26], sensoring [12,21,27], catalysis [12,28], biomedical [22,25,29], or optoelectronics [15,30].

Nevertheless, previous works have shown that the performance of the nanocomposites on most of these applications could be strongly influenced by the amount of filler incorporated in the polymer. For instance, Taghavimehr et al. [7] studied the effect of incorporating a wide range of ZnO nanoparticles on the rheological, mechanical, and electromagnetic properties, as well as on the foaming process of PS/ZnO samples, finding that ZnO contents about 40% provide the best performance. Pinto et al. [10] studied the effect of silver NPs (AgNPs) content on melamine (ME) foams (ranging from 0.2 to 18.6 wt.%) used as antibacterial filters for water treatment, demonstrating that contents about 9.6 wt.% are enough to obtain an excellent bactericide performance of the ME/Ag filters, while higher loads do not provide any enhancement and increased the risk of exceeding the safety threshold for the Ag^+^ ions release.

Consequently, it is essential to have straightforward approaches to determine the amount of nanoparticles on the nanocomposites. Currently, the most efficient characterization methods follow destructive approaches. Accurate determination of the nanoparticles load is often obtained by thermo gravimetric analysis (TGA) or induced couple plasma-optical emission spectroscopy (ICP-OES) [8,10,12,14]. Other less invasive methodologies are widely employed for the characterization of nanocomposites, such as transmission electron microscopy (TEM), high-resolution scanning electron microscopy (HRSEM), energy-dispersive X-ray (EDX), or X-ray photoelectron spectroscopy (XPS) [3,8,10,14,31,32]. However, these techniques have significant disadvantages. They usually require complex sample preparation (time-consuming), and in terms of the nanoparticles load determination, provide only qualitative information or quantitative elemental information limited to the surface of the nanocomposite [2]. On the contrary, non-invasive techniques commonly employed on the characterization of nanocomposites, such as Fourier transform infrared (ATR-FTIR), ultra-violet visible UV-vis, and Raman spectroscopies, are also employed to identify the presence of the nanoparticles, but present limitations to quantify their amount [21,31,33]. In addition, there are less conventional non-invasive techniques which can be employed on nanocomposites, such as colorimetry [31,32,33,34,35] and x-ray radiography, [9,36,37,38] which has not been previously tested with this aim.

On the one hand, colorimetry analysis can be carried out with inexpensive and portable instruments which allow the in situ testing of diverse materials working as sensors [32,33,34,35]. For instance, Wang et al. [32] reported the use of ZnO/ZnFe_2_O_4_/graphene foams as a sensing platform for colorimetric detection of hydroquinone, showing their potential for actual environmental pollutant analysis on river waters. In addition, there are a plenty of applications in which colorimetric detection technique can be employed, such as catalytic activity determination [32], moisture sensoring [31], HCl gas detection [33], or pH-response monitoring [35].

On the other hand, X-ray imaging has been employed to characterize nanocomposite porous materials in terms of their structure, phase dispersion (solid, gas, or fillers), or even to follow in situ the growth of polymeric foams and study the effect of the filler incorporation [9,36,37,38]. Santiago-Calvo et al. [9] employed X-ray radioscopy to study the effect of the incorporation of graphene oxide on rigid polyurethane foams in terms of the evolution of their relative density, cell size, and cell nucleation density during the foaming process. Also, Pardo-Alonso et al. [36] reported that the use of nanofillers (nanoclays and nanosilicas) produce a significant enhancement in the nucleation mechanism, reducing cell size and increasing cell density. Besides, Prade et al. [37] developed a method to study the orientation of the fibers in short fiber-reinforced polymer composites based on X-ray vector radiography, showing the potential of this technique for the non-invasive characterization of this kind of nanocomposites. In addition, Roels and Carmeliet [38] employed X-ray radiography to measure the process of moisture transport in cellular materials. They obtained accurate data on the moisture content and proved the applicability of the method on two different materials with different structures, homogeneous and heterogeneous.

Herein, facile non-invasive approaches based on colorimetry and X-ray radiography to determine the amount of noble metal NPs synthesized in porous polymer foams are presented. First, AgNPs were in situ synthesized in commercial porous melamine (ME) foams following the approach proposed by Pinto et al. [10]. A wide set of ME foams and AgNPs nanocomposites (ME/Ag) was obtained using two AgNO_3_ solutions (0.85 and 1.70 mg/mL) and different reaction times (from 1 to 7 days). Then, the obtained ME/Ag foams were analyzed by different approaches to determine the amount of AgNPs synthesized on the foams. In particular, two methodologies based on colorimetry and one on X-ray radiography were successfully tested. All these non-invasive approaches were calibrated using reference values previously reported by using a destructive ICP-based method [10], and provided accurate results on the entire set of ME/Ag foams.

## 2. Materials and Methods

### 2.1. Materials

Commercial melamine (ME) foams with high porosity (0.994), open pores, and mean pore size below 500 μm were kindly provided by Flexicel Industrial S.L. (Spain). Silver nitrate (AgNO_3_) and tetrahydrofuran (THF) were purchased from Scharlab S.L. (Spain). Deionized water was employed for the preparation of the silver nanoparticles (AgNPs) precursor solutions as well as for the rinsing of the foams.

### 2.2. Functionalization of the ME Foams

The in situ synthesis of silver nanoparticles (AgNPs) on the surface of pure melamine (ME) foams (samples 2 × 1 × 1 cm^3^) was achieved following the approach proposed by Pinto et al. [10] which provides a rather constant AgNPs distribution along the foams. The pure foams were immersed in 20 mL solutions with two different AgNO_3_ concentrations (0.85 and 1.70 mg/mL in a H_2_O/THF mixture (1:19 v:v)) and shook for different times ranging from 1 to 7 days. The AgNPs synthesis is expected to happen following a redox reaction involving the oxidation of –NH_2_ groups of the melamine and the reduction of the Ag^+^ ions ($3Ag^{+}NO_{3}^{-}+Melamine-NH_{2}+H_{2}O\;\to 3Ag^{0}+Melamine=N-OH+3H^{+}NO_{3}^{-}$) [10]. After the reaction time was reached, the samples were washed for five cycles, each cycle involved immersing the foam in 20 mL of distilled water, and shaking it for 2 min. Finally, all the samples were dried at room temperature.

### 2.3. Experimental Techniques

The structure and morphology of the ME foams, before and after the synthesis of the AgNPs were studied by scanning electron microscopy (SEM) (HITACHI FlexSEM 1000) after being coated with gold (10 nm) and by optical microscopy using a Microscopy EduBlue at 40×. SEM micrographs were obtained using an accelerating voltage of 10 kV and a working distance of 8 mm.

The determination of the amount of AgNPs synthesized (wt.%) into the foams was attempted by different methodologies, using the values previously reported by Pinto et al. [10] (measured by inductive couple plasma-optical emission spectroscopy (ICP-OES)) as reference values (see Section S1 in the Supplementary Information for more details). It should be noticed that only values for reaction times of 1, 3, 5, and 7 days were previously reported. Thermo gravimetric analysis (TGA) (SDTA851, Mettler Toledo) was performed with a heating ramp from 50 to 800 °C at a pace of 20 °C/min with nitrogen atmosphere (60 mL/min), followed by an isotherm at 800 °C for 15 min with air atmosphere (200 mL/min). Three samples of about 2 mg were extracted from different regions of each foam and analyzed by TGA, whereas the amount of AgNPs on the ME/Ag foams was estimated from the residue obtained for the ME and the ME/Ag foams (see Section S1 in the Supplementary Information for more details). Color measurement was carried out using a commercial color reader Colourpin SE (NCS Color AB, USA) using a standard illumination D65, 10° standard observer, and achieving a precision ΔE*_ab_ < 0.1. This color reader provides the color coordinates of the sample on the standard color space CIE L*a*b*, where there are tree axes orthogonal to each other: measurable lightness L* (values range from 0 (black) to 100 (white)); and two chromatic axes a* (values from green (negative) to red (positive)), and b* (goes from blue (negative) to yellow (positive)). The mean values and standard deviation of each coordinate were obtained from at least three measurements of each sample carried out both in external surfaces of the samples and internal surfaces exposed by cutting. The color differences between the pure ME foams and the obtained ME/Ag foams were evaluated in terms of the change of the luminosity (ΔL*, Equation (1)) and CIE L*a*b* color change (ΔE*_ab_, Equation (2)) which takes into account the three color coordinates.

(1) $$∆L^{*}=L^{*}{}_{ME/Ag}-L^{*}{}_{ME\;},\;\;$$

(2) $$∆E_{\;}^{*}{}_{ab}=\sqrt{{\left(∆L^{*}\right)}^{2}+{\left(a_{ME/Ag}^{*}-a_{ME}^{*}\right)}^{2}+{\left(b_{ME/Ag}^{*}-b_{ME}^{*}\right)}^{2}}$$

X-ray radiographs were obtained using a constructed X-ray equipment consisting of a low energy micro-focus X-ray source (L10101, Hamamatsu. Voltage: 20–100 kV, Current: 0–200 μA), and a high sensitivity flat panel detector (C7940DK-02, Hamamatsu. 2240 × 2344 pixels, 50 μm pixel size) [9,36,39]. Sections with thickness about 2.8–2.9 mm were taken from the foams to perform these measurements. The radiographs were acquired with a tube voltage and current fixed at (40 kV, 170 µA) and an exposure time of 1500 ms. In order to enhance the contrast, each image was the result of integrating four consecutive images. The linear attenuation coefficient, µ (m^−1^), of each foam has been calculated after applying logarithmic conversion to Beer-Lambert’s attenuation law (Equation (3)). This equation relates to the intensity transmitted through the material, *I*, with the initial X-ray beam intensity, *I*_0_, and the sample’s thickness, *t*.

(3) $$\mu =\frac{1}{t}\cdot ln\left(\frac{I_{0}}{I}\right)$$

It is known that the attenuation of a two-phase system is a linear combination of that of every present phase. In particular, the attenuation coefficient of a material (µ) is a function of the number concentration, C (mol/m^3^), of the present species and their X-ray cross-section, σ (m^2^) (Equation (4)). Since the ME/Ag samples were obtained from the same initial foam without significant alteration of the polymer matrix, the value of *µ_ME_* will be the same for the treated foams and the reference pure foam. Meanwhile, the second term in Equation (4) (*µ_Ag_*) will change depending on the amount of AgNPs present on the ME/Ag foams.

(4) $$\mu =\mu_{ME}+\mu_{Ag}=\sigma_{ME}*C_{ME}+\sigma_{Ag}*C_{Ag}$$

Thus, it should be possible to obtain the attenuation coefficient because of the sole presence of different amounts of AgNPs (*µ_Ag_*) for every foam from the measured *µ* and *µ_ME_* values [40].

Finally, the comparison between the different techniques employed was carried out by calculating the mean squared error (MSE) of the values provides for each method with respect to the trends obtained from the ICP-OES values employed as a reference (Equation (5)).

(5) $$MSE=\frac{1}{n}\overset{n}{\underset{i=1}{\sum }}{\left(Y_{i}-{\hat{Y}}_{i}\right)}^{2}$$

where *n* is the number of samples, *Y_i_* is the value obtained from the different methods and *Ŷ_i_* is the value previously provided by the ICP-OES measurements [10].

## 3. Results and Discussion

### 3.1. Characterization of the ME and ME/Ag Foams

**Figure 1:**
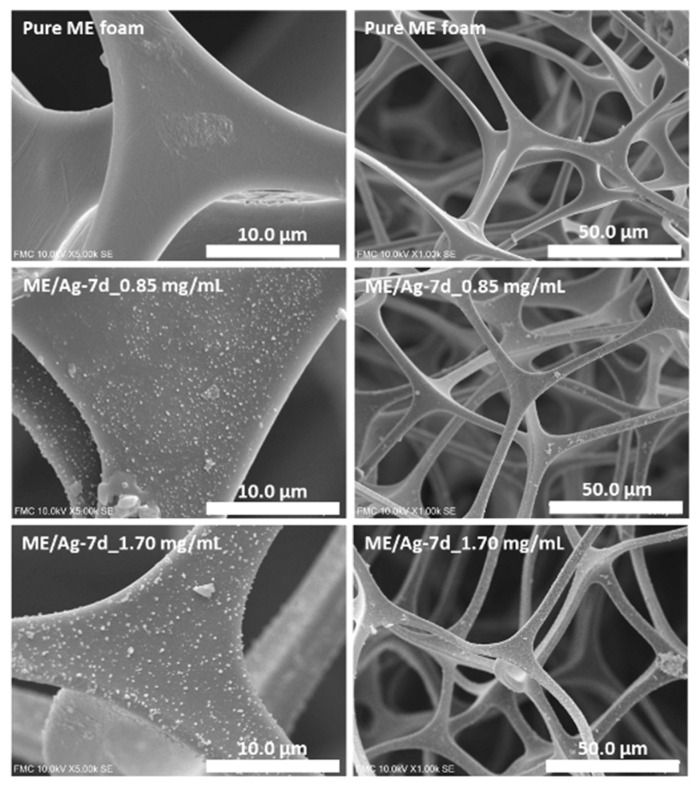
Scanning electron microscopy (SEM) micrographs of the untreated surface of melamine (ME) (Pure) and ME/Ag obtained after 7 day of reaction times using 0.85 and 1.70 mg/mL as precursor solution, showing a homogeneous distribution of the AgNPs along the strut in both lower and high magnification.

It is well-known that the amount of NPs transferred to the foam following this in situ synthesis approach depends mainly on the reaction time and the concentration of precursors. Therefore, in order to have an appropriate set of samples for this study, ME/Ag foams were prepared using two different AgNO_3_ concentrations (0.85 and 1.70 mg/mL) and seven reaction times for each concentration (1 to 7 days). These samples are expected to provide a wide range of AgNPs loads in which the proposed techniques to determine the amount of NPs (wt.%) can be validated. In the first step, the porous structure of the produced foams was analyzed by SEM microscopy (Figure 1).

As previously reported by Pinto et al. [10], no significant differences were observed in the porous structure of the ME foams after being immersed in the AgNO_3_ solutions with different concentrations (0.85 and 1.70 mg/mL) for several days (from 1 to 7). In fact, porous structure of the functionalized foams presents no changes even after 7 days of reaction time, independently of the concentration of the precursor solution (Figure 1, more details in the Supplementary Information, Section S2). Moreover, focusing on the ME surfaces, it was possible to identify in the functionalized foams the presence of aggregates of AgNPs with sizes around 200–300 nm (Figure 1, Figures S2–S4). However, previous studies proved that, in addition to these aggregates, these surfaces are homogeneously covered by individual AgNPs with average sizes below 10 nm [10].

**Figure 2:**
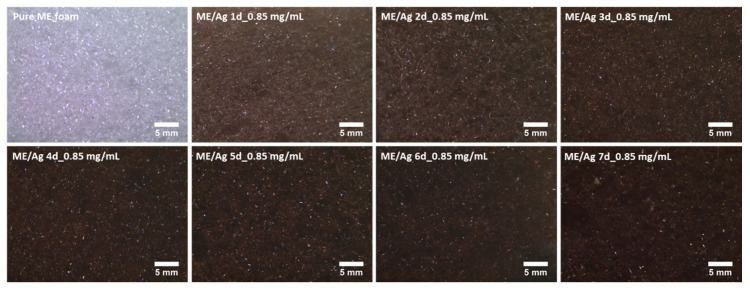
Optical micrographs (40×) of the untreated ME (Pure) and functionalized ME/Ag using 0.85 mg/mL as solution precursor, showing a homogeneously distributed color along the foams, darkening as the reaction time was increased.

**Figure 3:**
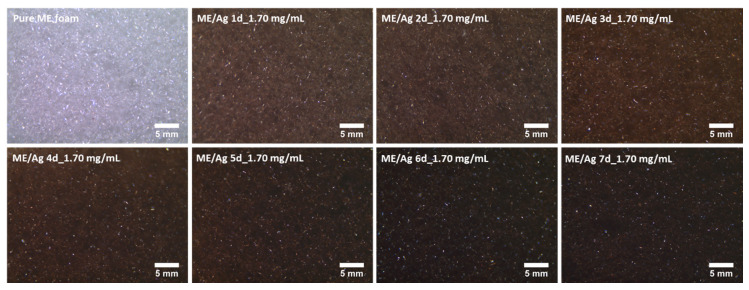
Optical micrographs (40×) of the untreated ME (Pure) and functionalized ME/Ag using 1.70 mg/mL as solution precursor, showing a homogeneously distributed color along the foams, darkening as the reaction time was increased.

The influence of the preparation parameters of ME/Ag foams and the AgNPs distribution was also studied by optical microscopy (Figure 2 and Figure 3). Regardless of the precursor concentration employed, the functionalization of ME foams with the AgNO_3_ solutions induced an evident change in the color of the samples, starting after the first day. The color changes from light gray for the pure ME foams to light brown on the ME/Ag foams obtained after one day. This brown color is a clear indication of the presence of AgNPs [10].

**Figure 4:**
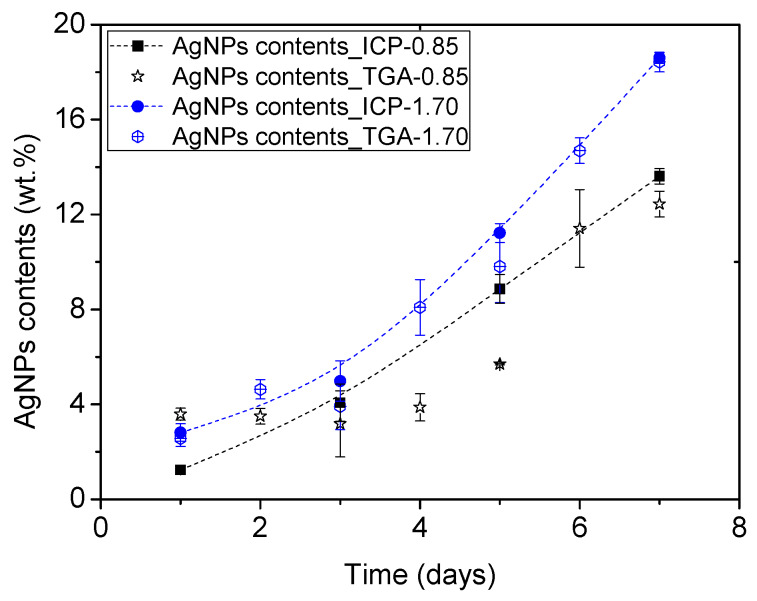
AgNPs content (wt.%) measured by thermo gravimetric analysis (TGA) and induced couple plasma-optical emission spectroscopy (ICP-OES) [10] in the functionalized ME/Ag foams obtained after different reaction times in two concentrations of AgNO_3_.

In addition, optical micrographs of the entire set of samples (Figure 3 and Figure 4) show that the foams become darker as the reaction time increases. These micrographs, showing a large area of the samples, prove that the functionalized foams present a quite homogeneous color, which suggests that a homogeneous AgNPs coating was obtained. Furthermore, it is known that the amount of NPs increases with the reaction time; therefore, the observed progressive change in color is a qualitative indication of the relationship between both magnitudes.

### 3.2. TGA Study of ME/Ag Foams

First, the amount of Au transferred to ME/Ag foams was attempted to be determined by TGA analysis, a conventional and destructive method for this kind of characterization (see Section S1 in the Supplementary Information for more details). Figure 4 shows the obtained results compared with the results previously published using ICP-OES analysis [10]. A wide range of contents (1 to 18 wt.%) is obtained indicating that the preparation process was successful in producing materials with enough differences in composition. In general, the samples with lower content of NPs (less than 5 wt.%) do not follow the expected trends. At the same time, at medium and high contents, the data adjust better to the trends, although with significant deviations regarding the values obtained in several cases. These discrepancies can have two origins. On the one hand, the TGA technique has an experimental error of 1% (according to the technical specifications of the measurement system), which implies errors of up to 33% in the estimation of the AgNPs contents at low values (TGA residues about 11 wt.%), while only about 6% at the higher contents (TGA residues about 25 wt.%). On the other hand, the determination of the AgNPs amount from the TGA residue was performed assuming a constant ME residue after the TGA program (about 8 wt.%), being not possible to discard that in the presence of AgNPs the ME degradation behaves differently.

### 3.3. Colorimetry Study of ME/Ag Foams

As shown in Figure 2 and Figure 3, the immersion of the ME foams into AgNO_3_ solutions induced a color change on the surface of the foams, which seemed to be related to the amount of AgNPs present on the foams. Thus, a colorimetric analysis was performed to determine accurately the color coordinates in each foam. Then, this information can be related to the preparation conditions (i.e., reaction time and precursor solution concentration) to check if it allows monitoring the amount of AgNPs synthesized on the foams. Average values and standard deviations of the colorimetric measurements are shown in Table 1.

As a first observation, there is a direct relationship between the coordinate L with the reaction time. In fact, ME foams present the higher values of L* (Luminosity) corresponding to their light gray color. Then, this value decreases considerably by about 30.70 and 35.44% after being immersed for one day in 0.85 and 1.70 mg/mL AgNO_3_ solutions, respectively. Moreover, after 7 days of reaction time, the values decrease by about 61.30 and 62.90%. Therefore, the luminosity (L) seems to be quite sensible to the presence of AgNPs, providing noticeable differences for AgNPs contents ranging from about 1 to 18 wt.% regarding the pure ME foams.

**Figure 5:**
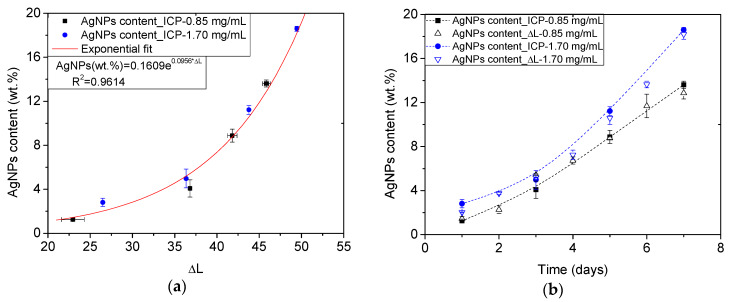
Calibration curve relating the AgNPs content (wt.%) reference values obtained by ICP-OES [10] and the ΔL* measured on the ME/Ag foams. The obtained calibration equation is shown in an inset (**a**). AgNPs content (wt.%) of all ME/Ag foams calculated from the measured ΔL* and using the previous calibration. Trends of the reference values from ICP-OES measurements are shown in dotted lines for comparison purposes (**b**).

Thus, it was tested if the luminosity variation (ΔL*) term, obtained following Equation (1), might provide information about the amount of AgNPs. First, the obtained ΔL* values from ME/Ag foams obtained after 1, 3, 5, or 7 days of reaction time were related to their known amount of AgNPs (previously determined by ICP-OES elsewhere [10]) (Figure 5a). It was found that these values follow a clear trend, which can be accurately fitted using an exponential expression (R^2^ = 0.96). It should be noticed that this expression is not related to the precursor solution or reaction time employed, but only to the amount of AgNPs of the foam. Therefore, it can be applied as a general equation to determine the amount of AgNPs synthesized on the ME foams using both 0.85 and 1.70 mg/mL precursor solutions and any reaction time (Figure 5b).

The obtained values of the AgNPs content (wt.%) from the ΔL* measurements shown in Figure 5b were in good agreement with the known trends for both concentrations along with the entire time range.

**Figure 6:**
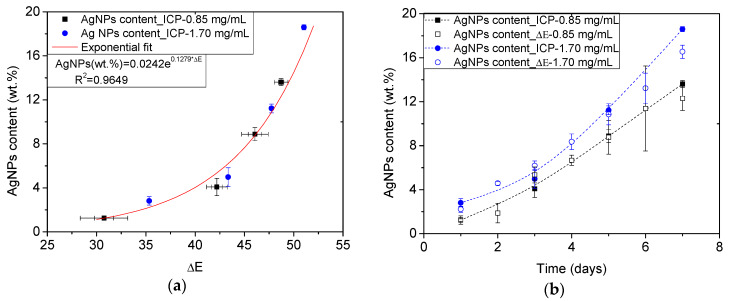
Calibration curve relating the AgNPs content (wt.%) reference values obtained by ICP-OES [10] and the ΔE*_ab_ measured on the ME/Ag foams. The obtained calibration equation is shown in an inset (**a**). AgNPs content (wt.%) of all ME/Ag foams calculated from the measured ΔE*_ab_ and using the previous calibration. Trends of the reference values from ICP-OES measurements are shown in dotted lines for comparison purposes (**b**).

However, the previous approach only considers one of the color coordinates provided by the colorimetry measurements (Table 1). In order to check if the measurement method can be improved by incorporating the information of the three color coordinates, the ΔE*_ab_ (Equation (2)) was used following the same procedure and retaking the pure foams as reference. Figure 6a shows the exponential fit (R^2^ = 0.97) of the values of ΔE*_ab_ related to the reference values of AgNPs. Yet, it is possible to use the obtained exponential expression to calculate the amount of AgNPs synthesized on the ME foams during the ME/Ag foams preparation (Figure 6b). Once again, the obtained values using this colorimetric approach provided AgNPs content values within the expected trends.

Therefore, it was found that both approaches involving colorimetry measurements provided values for the load of AgNPs in ME foams that comply with the expected trends, independently of the preparation parameters of the foams. Accordingly, this method proved to be reliable for the determination of the amount of AgNPs, with the additional advantages of being a non-invasive, macroscopic, inexpensive, fast, and simple measurement method.

### 3.4. X-ray Study of the ME/Ag Foams

**Figure 7:**
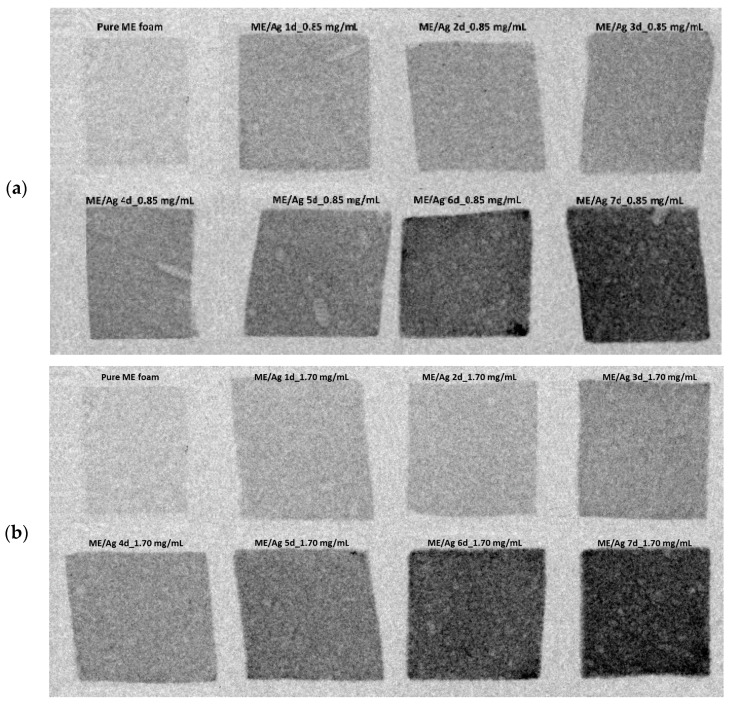
X-ray radioscopy of the untreated ME (pure) and functionalized ME/Ag using 0.85 mg/mL (**a**) and 1.70 mg/mL (**b**) as solution precursor for different reaction times from 1 to 7 days.

Figure 7 shows the acquired radiographs of the ME and the ME/Ag foams after being introduced in two solutions of AgNO_3_ with different concentrations, 0.85 mg/mL and 1.70 mg/mL, for reaction times ranging from 1 to 7 days (Figure 7a,b respectively). In both figures, a decrease in the foams’ X-ray transmissivity is detected with increasing reaction time. Considering that the thickness of the samples was kept practically constant (2.88 ± 0.16 mm) the decrease in X-ray transmissivity can only be attributed to a change in the foams’ attenuation coefficient because of the synthesized AgNPs on the surface of the ME foams. Besides, in the radiographs, some features of the cellular structure can be observed, such as the presence of large pores (in the millimetric range).

It is possible to detect how there was nearly total X-ray transmissivity by the pure ME foams prior to its immersion in the AgNO_3_ solutions. Melamine is a lightweight organic polymer; it is composed of elements with few electrons in their nucleus, such as nitrogen, hydrogen, and carbon. For X-rays, the probability of being attenuated correlates strongly with the number of electrons of an element (i.e., the atomic number Z). Therefore, metals, such as silver, induce strong X-ray attenuation, whereas lighter elements, such as hydrogen, attenuate weakly [41].

**Figure 8:**
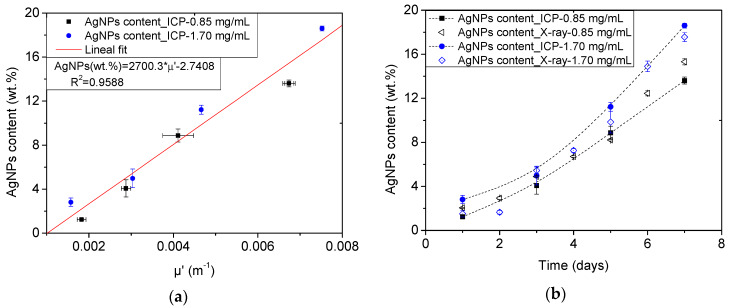
Calibration curve relating the AgNPs content (wt.%) reference values obtained by ICP-OES [10] and the μ’ measured on the ME/Ag foams. The obtained calibration equation is shown in an inset (**a**). AgNPs content (wt.%) of all ME/Ag foams calculated from the measured μ’ and using the previous calibration. Trends of the reference values from ICP-OES measurements are shown in dotted lines for comparison purposes (**b**).

In order to test whether the variation in the attenuation coefficient could be used as input to determine the silver content incorporated during the in situ synthesis, a calibration trend is sought by relating the obtained attenuation values to the reference AgNPs values (Figure 8a). A linear relationship was found, revealing that with increasing content of Ag, the attenuation of X-rays by the samples increased too (R^2^ = 0.96). In Figure 8b, it is possible to observe the evolution of the AgNPs content as obtained from the attenuation coefficient of the foams. Despite slight deviations in the absolute value, it can be detected how the increase in AgNPs contents determined with the use of X-ray imaging follows the trends observed with ICP-OES.

### 3.5. Accuracy of the Proposed Approaches

**Figure 9:**
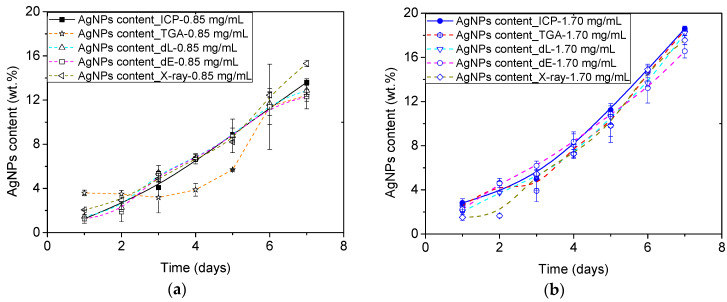
AgNPs contents of ME/Ag foams as a function of the reaction time using 0.85 mg/mL (**a**) and 1.70 mg/mL (**b**) as solution precursor.

AgNPs contents on the obtained ME/Ag foams have been determined using four different characterization methods: a conventional destructive TGA analysis, two approaches based on colorimetry, and one on X-ray radiography. Figure 9 and Table S2 summarize all the obtained results with the different techniques from the set of ME/Ag foams obtained from AgNO_3_ concentrations of 0.85 and 1.70 mg/mL, (Figure 9a,b, respectively).

As abovementioned, the three non-invasive approaches accurately reproduce the evolution of the AgNPs content with time previously found by ICP-OES results, independently of the AgNO_3_ concentration employed [10]. On the contrary, the result provided by TGA, seems to be not accurate for AgNPs contents below 5–8 wt.%.

A more detailed comparative analysis of these techniques is presented in Table 2, where the mean squared errors (Equation (5)) of each technique with respect to the trend of the ICP-OES values, are presented. As expected, the TGA analysis is found to be the less accurate (MSE = 2.05), while the proposed non-invasive approaches present similar values about or below 1. It should be noticed that according to these results the most accurate approach is the measurement of the changes on the luminescence (L*) color coordinate, which is also the more simple, quick, and inexpensive procedure proposed.

Therefore, the proposed non-invasive characterization approaches based on both colorimetry and X-ray radiography have been proved to be accurate on the determination of the AgNPs content of the ME/Ag foams. As these approaches rely on simple physical principles, it can be expected that their use could be extended to other nanocomposites when the differences between the polymer and the nanofiller are significative in terms of their color or X-ray absorbance. Thus, these techniques are promising approach for the characterization, production monitoring, or quality control of polymer nanocomposites incorporating noble metal nanoparticles (e.g., Au, Pd, Ag), which generally shown characteristic intense colors and present a higher X-ray attenuation than polymers [10,23].

## 4. Conclusions

This work developed novel non-invasive procedures for the study and characterization of the filler load on polymer nanocomposites. In particular, two approaches based on colorimetric analysis and one on X-ray radiography were successfully tested on a wide set of ME/Ag foams obtained by the in situ synthesis of Ag NPs on melamine (ME) foams.

On the one hand, the colorimetry-based techniques take advantage of the color change induced on the ME/Ag foams by the presence of AgNPs, which turn the light gray ME foams into brown tonalities that darkens with increasing amounts of AgNPs. A direct relationship between the luminosity and the overall color change of a reference set of ME/Ag samples and their known AgNPs load was found, allowing to obtain calibration equations relating these values. From these equations, it was possible to determine the load of the entire set of ME/Ag foams, finding an excellent agreement between those values and the known trends for the reference ME/Ag foams.

On the other hand, the differences on the X-ray absorption coefficients between the polymer matrix and the filler, in this case AgNPs, also allows the quantification of the AgNPs load by X-ray radiography. It was possible to calculate the absorption coefficients of the reference set of ME/Ag foams and relate them to their AgNPs load, obtaining again a calibration equation which accurately reproduce the expected trends for the entire set of ME/Ag samples.

In addition, a conventional destructive technique such as TGA was employed for comparison purposes. It was found that for the ME/Ag samples this technique provides less accurate results than those obtained with the non-invasive techniques proposed, as the presence of ME residue even at very-high temperatures, and the possible interaction between the ME and AgNPs during the ME degradation, make this method inaccurate for low AgNPs contents.

Therefore, the proposed non-invasive procedures proved to be suitable and facile approaches for the determination of the AgNPs load on the ME/Ag nanocomposite foams. Moreover, the colorimetry-based approaches proved to be the most accurate, while this technique is also inexpensive and can be easily carried out in situ. Also, these techniques could be applied to other nanocomposites with proper features in terms of color or X-ray attenuation differences.

## Figures and Tables

**Table 1:** Colorimetry CIE L*a*b* coordinates obtained for the pure ME foam and ME/Ag foams produced using 0.85 and 1.70 mg/mL precursor solutions as well as different reaction times from 1 to 7 days.

Samples	Reaction Time (days)	Precursor Solution (mg/mL)	L*	a*	b*
Me_Pure	--		74.78 ± 0.72	−1.33 ± 0.31	−3.93 ± 0.44
Me_1d-0.85	1	0.85	51.82 ± 2.06	7.93 ± 0.53	14.32 ± 2.43
Me_2d-0.85	2	0.85	47.10 ± 2.30	8.14 ± 1.12	13.39 ± 3.54
Me_3d-0.85	3	0.85	37.97 ± 0.85	8.65 ± 1.35	14.14 ± 0.62
Me_4d-0.85	4	0.85	35.71 ± 0.23	9.13 ± 0.52	13.26 ± 0.38
Me_5d-0.85	5	0.85	32.97 ± 0.86	9.54 ± 0.46	12.05 ± 1.77
Me_6 d-0.85	6	0.85	29.95 ± 1.67	10.90 ± 1.64	8.56 ± 2.47
Me_7d-0.85	7	0.85	28.94 ± 0.28	8.11 ± 0.79	9.61 ± 0.68
Me_1d-1.70	1	1.70	48.28 ± 0.65	9.10 ± 0.93	17.00 ± 1.18
Me_2d-1.70	2	1.70	41.87 ± 0.41	9.80 ±0.38	17.84 ± 0.48
Me_3d-1.70	3	1.70	38.42 ± 0.65	9.86 ± 0.85	16.83 ± 0.47
Me_4d-1.70	4	1.70	34.94 ± 0.11	10.14 ± 0.36	15.30 ± 0.20
Me_5d-1.70	5	1.70	30.99 ± 0.16	9.56 ± 0.23	11.66 ± 0.06
Me_6d-1.70	6	1.70	28.33 ± 0.95	8.12 ± 1.08	9.59 ± 0.40
Me_7d-1.70	7	1.70	27.74 ± 0.29	8.33 ± 0.97	8.31 ± 1.70

**Table 2:** Mean squared error (Equation (5)) of the different values obtained for the AgNPs contents of ME/Ag foams using 0.85 and 1.70 mg/mL as solution precursor, as well as the different reaction times.

Samples	TGA-ICP	ΔL-ICP	ΔE-ICP	X-ray-ICP
ME/Ag	2.05	0.31	0.85	1.03

## Supplementary Materials

The following are available online at https://www.mdpi.com/2073-4360/12/5/996/s1. Additional experimental details (ICP and TGA), and results from the SEM, atomic force microscopy (AFM), and colorimetry characterization of the ME/Ag foams. Summary of all the obtained results.

## References

[1] Zhao X., Lv L., Pan B., Zhang W., Zhang S., Zhang Q. (2011). Polymer-supported nanocomposites for environmental application: A review. Chem. Eng. J..

[2] Barroso-Solares S., Merillas B., Cimavilla-Román P., Rodriguez-Perez M.A., Pinto J. (2020). Enhanced nitrates-polluted water remediation by polyurethane/sepiolite cellular nanocomposites. J. Clean. Prod..

[3] Calcagnile P., Fragouli D., Mele E., Ruffilli R., Athanassiou A. (2014). Polymeric foams with functional nanocomposite cells. RSC Adv..

[4] Tamayo L., Palza H., Bejarano J., Zapata P.A. (2019). Polymer Composites With Metal Nanoparticles.

[5] Cataldi P., Ceseracciu L., Athanassiou A., Bayer I.S. (2017). Healable Cotton-Graphene Nanocomposite Conductor for Wearable Electronics. ACS Appl. Mater. Interfaces.

[6] Deng C.H., Gong J.L., Zhang P., Zeng G.M., Song B., Liu H.Y. (2017). Preparation of melamine sponge decorated with silver nanoparticles-modified graphene for water disinfection. J. Colloid Interface Sci..

[7] Taghavimehr M., Navid Famili M.H., Shirsavar M.A. (2020). Effect of nanoparticle network formation on electromagnetic properties and cell morphology of microcellular polymer nanocomposite foams. Polym. Test..

[8] Zhou S., Hao G., Zhou X., Jiang W., Wang T., Zhang N., Yu L. (2016). One-pot synthesis of robust superhydrophobic, functionalized graphene/polyurethane sponge for effective continuous oil-water separation. Chem. Eng. J..

[9] Santiago-Calvo M., Pérez-Tamarit S., Cimavilla-Román P., Blasco V., Ruiz C., París R., Villafañe F., Rodríguez-Pérez M.Á. (2019). X-ray radioscopy validation of a polyol functionalized with graphene oxide for producing rigid polyurethane foams with improved cellular structures. Eur. Polym. J..

[10] Pinto J., Magrì D., Valentini P., Palazon F., Heredia-Guerrero J.A., Lauciello S., Barroso-Solares S., Ceseracciu L., Pompa P.P., Athanassiou A. (2018). Antibacterial Melamine Foams Decorated with in Situ Synthesized Silver Nanoparticles. ACS Appl. Mater. Interfaces.

[11] Lei Z., Zhang G., Ouyang Y., Liang Y., Deng Y., Wang C. (2017). Simple fabrication of multi-functional melamine sponges. Mater. Lett..

[12] Charara M., Luo W., Saha M.C., Liu Y. (2019). Investigation of Lightweight and Flexible Carbon Nanofiber/Poly Dimethylsiloxane Nanocomposite Sponge for Piezoresistive Sensor Application. Adv. Eng. Mater..

[13] Almeida J.C., Cardoso C.E.D., Pereira E., Freitas R., Gonçalves G.A.B., Marques P. (2019). Toxic Effects of Metal Nanoparticles in Marine Invertebrates. Nanostructured Materials for Treating Aquatic Pollution.

[14] Zeng J., Xu P., Chen G., Zeng G., Chen A., Hu L., Huang Z., He K., Guo Z., Liu W. (2019). Effects of silver nanoparticles with different dosing regimens and exposure media on artificial ecosystem. J. Environ. Sci. (China).

[15] Zada A., Muhammad P., Ahmad W., Hussain Z., Ali S., Khan M., Khan Q., Maqbool M. (2020). Surface Plasmonic-Assisted Photocatalysis and Optoelectronic Devices with Noble Metal Nanocrystals: Design, Synthesis, and Applications. Adv. Funct. Mater..

[16] Dauthal P., Mukhopadhyay M. (2016). Noble Metal Nanoparticles: Plant-Mediated Synthesis, Mechanistic Aspects of Synthesis, and Applications. Ind. Eng. Chem. Res..

[17] Barroso-Solares S., Pinto J., Fragouli D., Athanassiou A. (2018). Facile oil removal from water-in-oil stable emulsions using PU foams. Materials.

[18] Mohd Zaini N.A., Ismail H., Rusli A. (2017). A Short Review on Sepiolite-Filled Polymer Nanocomposites. Polym. Plast. Technol. Eng..

[19] Cavallaro G., Lazzara G., Milioto S., Parisi F., Evtugyn V., Rozhina E., Fakhrullin R. (2018). Nanohydrogel Formation within the Halloysite Lumen for Triggered and Sustained Release. ACS Appl. Mater. Interfaces.

[20] Lazzara G., Cavallaro G., Panchal A., Fakhrullin R., Stavitskaya A., Vinokurov V., Lvov Y. (2018). An assembly of organic-inorganic composites using halloysite clay nanotubes. Curr. Opin. Colloid Interface Sci..

[21] Wu M., Zhang C., Ji Y., Tian Y., Wei H., Li C., Li Z., Zhu T., Sun Q., Man B. (2020). 3D Ultrasensitive Polymers-Plasmonic Hybrid Flexible Platform for In-Situ Detection. Polymers.

[22] Jiang X., Du B., Huang Y., Zheng J. (2018). Ultrasmall noble metal nanoparticles: Breakthroughs and biomedical implications. Nano Today.

[23] Zhang Z., Wang H., Chen Z., Wang X., Choo J., Chen L. (2018). Plasmonic colorimetric sensors based on etching and growth of noble metal nanoparticles: Strategies and applications. Biosens. Bioelectron..

[24] Pinto J., Athanassiou A., Fragouli D. (2018). Surface modification of polymeric foams for oil spills remediation. J. Environ. Manag..

[25] Kim H.S., Lee D.Y. (2018). Near-infrared-responsive cancer photothermal and photodynamic therapy using gold nanoparticles. Polymers.

[26] Gupta R., Kulkarni G.U. (2011). Removal of organic compounds from water by using a gold nanoparticle-poly(dimethylsiloxane) nanocomposite foam. ChemSusChem.

[27] Wang J., Hou L., Yan K., Zhang L., Yu Q.J. (2018). Polydopamine nanocluster decorated electrospun nanofibrous membrane for separation of oil/water emulsions. J. Memb. Sci..

[28] Rodrigues T.S., Da Silva A.G.M., Camargo P.H.C. (2019). Nanocatalysis by noble metal nanoparticles: Controlled synthesis for the optimization and understanding of activities. J. Mater. Chem. A.

[29] Azharuddin M., Zhu G.H., Das D., Ozgur E., Uzun L., Turner A.P.F., Patra H.K. (2019). A repertoire of biomedical applications of noble metal nanoparticles. Chem. Commun..

[30] Pérez-Jiménez L.E., Solis-Cortazar J.C., Rojas-Blanco L., Perez-Hernandez G., Martinez O.S., Palomera R.C., Paraguay-Delgado F., Zamudio-Torres I., Morales E.R. (2019). Enhancement of optoelectronic properties of TiO2 films containing Pt nanoparticles. Results Phys..

[31] Song Q., Li M., Wang L., Ma X., Liu F., Liu X. (2019). Mechanism and optimization of electrochemical system for simultaneous removal of nitrate and ammonia. J. Hazard. Mater..

[32] Wang X., Zhao M., Song Y., Liu Q., Zhang Y., Zhuang Y., Chen S. (2019). Synthesis of ZnFe2O4/ZnO heterostructures decorated three-dimensional graphene foam as peroxidase mimetics for colorimetric assay of hydroquinone. Sens. Actuators B Chem..

[33] Mergu N., Kim H., Heo G., Son Y.A. (2020). Fabrication and topochemically controlled diacetylene-based polymer and its colorimetric application toward HCl detection. Dye. Pigment..

[34] Mergu N., Kim H., Ryu J., Son Y.A. (2020). A simple and fast responsive colorimetric moisture sensor based on symmetrical conjugated polymer. Sens. Actuators B Chem..

[35] Ko Y., Jeong H.Y., Kwon G., Kim D., Lee C., You J. (2020). pH-responsive polyaniline/polyethylene glycol composite arrays for colorimetric sensor application. Sens. Actuators B Chem..

[36] Pardo-Alonso S., Solórzano E., Rodriguez-Perez M.A. (2013). Time-resolved X-ray imaging of nanofiller-polyurethane reactive foam systems. Colloids Surf. A Physicochem. Eng. Asp..

[37] Prade F., Schaff F., Senck S., Meyer P., Mohr J., Kastner J., Pfeiffer F. (2017). Nondestructive characterization of fiber orientation in short fiber reinforced polymer composites with X-ray vector radiography. NDT E Int..

[38] Roels S., Carmeliet J. (2006). Analysis of moisture flow in porous materials using microfocus X-ray radiography. Int. J. Heat Mass Transf..

[39] Solórzano E., Pinto J., Pardo S., Garcia-Moreno F., Rodriguez-Perez M.A. (2013). Application of a microfocus X-ray imaging apparatus to the study of cellular polymers. Polym. Test..

[40] Melnichenko Y.B., Wignall G.D., Cole D.R., Frielinghaus H. (2006). Adsorption of supercritical CO_2_ in aerogels as studied by small-angle neutron scattering and neutron transmission techniques. J. Chem. Phys..

[41] Bracewell B.L., Veigele W.J. (1971). Tables of X-ray Mass Attenuation Coefficients for 87 Elements at Selected Wavelengths. Developments in Applied Spectroscopy.

