# Supplementary material for: Non-Invasive Approaches for the Evaluation of the Functionalization of Melamine Foams with In-Situ Synthesized Silver Nanoparticles
Source: Polymers (Basel). 2020 Apr 25;12(5):996. doi: 10.3390/polym12050996 (PMC7285167; doi:10.3390/polym12050996)
Supplement: Supplementary file 1 [file polymers-12-00996-s001.pdf]

polymers

MDPI

Supplementary Information

Non-Invasive Approaches for the Evaluation of the Functionalization of Melamine Foams with In Situ Synthesized Silver Nanoparticles

Suset Barroso-Solares [1,2,3,*], Paula Cimavilla-Roman [1], Miguel Angel Rodriguez-Perez [1,2,] and Javier Pinto [1,2]

[1] Cellular Materials Laboratory (CellMat), Condensed Matter Physics Department, University of Valladolid, 47011 Valladolid, Spain; paulacimavilla@fmc.uva.es

[2] BioecoUVA Research Institute, University of Valladolid, 47011, Valladolid, Spain; marrod@fmc.uva.es (M.A.R.-P.); jpinto@fmc.uva.es (J.P.)

[3] Group UVASENS, Escuela de Ingenierías Industriales, University of Valladolid, Paseo del Cauce, 59, Valladolid, 47011, Spain

* Correspondence: sbarroso@fmc.uva.es.

S1. Determination of the AgNPs Content of ME/Ag Foams

S1.1. ICP-OES

ME/Ag foams obtained following the same production route and using reaction times of 1, 3, 5, and 7 days were characterized by ICP-OES in a previous work [10]. These results are employed as reference values in this work.

In that work the sample preparation for ICP-OES was carried out by digesting 5 mg of the ME/Ag foams in 2.5 mL of nitric acid using a microwave digestion system (MARS Xpress, CEM). While the solid degradation was performed at 180 °C for 15 min. Then, the samples were diluted with Milli-Q water up to 25 mL and filtered using polytetrafluoroethylene syringe filters (15 mm, pore size 0.45 μm, Sartorius).

S1.2. TGA

Figure S1 shows the TGA curves obtained for the pure ME foam and a ME/Ag foam obtained after 4 days immersed in a 1.70 mg/mL Ag precursor solution. A clear difference is found in the remaining residue at the end of the heating program.

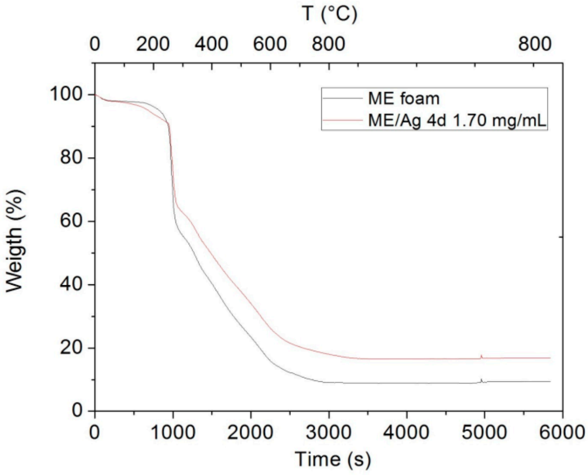

Figure S1. TGA curves obtained for the pure ME foam and a ME/Ag foam obtained after 4 days immersed in a 1.70 mg/mL Ag precursor solution.

Average values from different areas of each sample and different samples produced by the same procedure were employed to estimate the AgNPs load of the ME/Ag foams. In particular, the AgNPs (AgNPs wt.%) and ME (ME wt.%) contents can be estimated from the total ME/Ag residue ($TGA_{ME/Ag}$) and the pure ME (TGA_{ME} = 7.9 %) and Ag residues (TGA_{Ag} = 100 %) as follows:

$$TGA_{ME/Ag} = ME\ wt.\% \times \frac{TGA_{ME}}{100} + AgNPs\ wt.\% \times \frac{TGA_{Ag}}{100}$$

$$TGA_{ME/Ag} = ME\ wt.\% \times \frac{TGA_{ME}}{100} + (100 - ME\ wt.\%) \times \frac{TGA_{Ag}}{100}$$

$$TGA_{ME/Ag} = ME\ wt.\% \times \frac{TGA_{ME}}{100} + 100 - ME\ wt.\%$$

$$TGA_{ME/Ag} - 100 = ME\ wt.\% \times (\frac{TGA_{ME}}{100} - 1)$$

$$ME\ wt.\% = \frac{TGA_{ME/Ag} - 100}{\frac{TGA_{ME}}{100} - 1} \text{ and } AgNPs\ wt.\% = 100 - \frac{TGA_{ME/Ag} - 100}{\frac{TGA_{ME}}{100} - 1}$$

As an example, ME foams left an average residue of 7.9 wt.%, while ME/Ag foams obtained after 4 days immersed in a 1.70 mg/mL Ag precursor solution left an average residue of 15.4 wt.%.

$$ME\ wt.\% = \frac{15.4 - 100}{\frac{7.9}{100} - 1} = 91.9\ wt.\% \text{ and } AgNPs\ wt.\% = 8.1\ wt.\%$$

S2. In Situ Synthesis of AgNPs on the Surface of ME Foams (ME/Ag)

Figure S2 shows the pure ME and ME/Ag foams obtained after different reaction times (from 1 to 7 days) using 0.85 mg/mL as solution precursor. While Figure S3 shows the pure ME and ME/Ag foams obtained after different reaction times (from 1 to 7 days) using 1.70 mg/mL as precursor solution. For both precursor solutions, an increase of presence of AgNPs aggregates is observed on the surface of the struts of the functionalized foams with the increase of the reaction time.

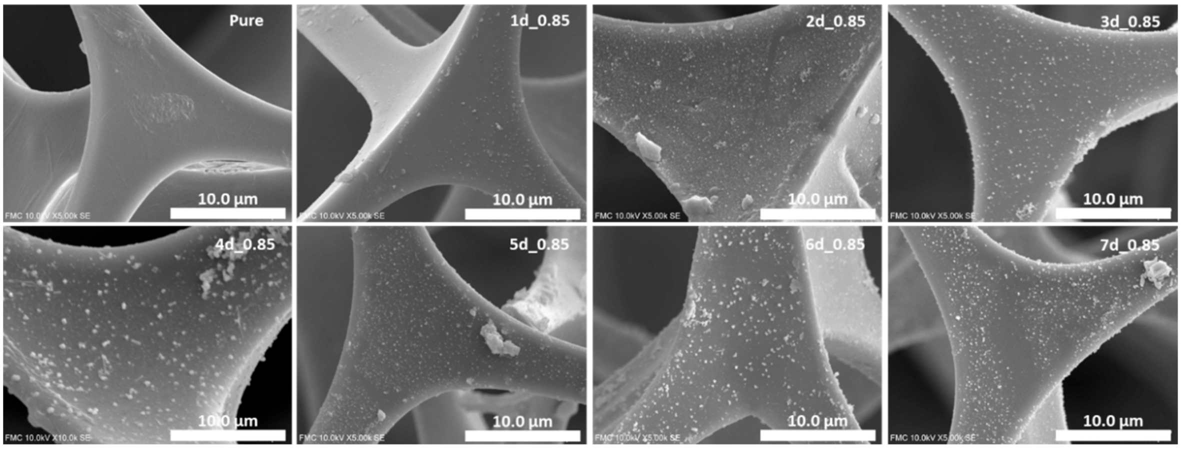

Figure S2. SEM micrographs of the untreated ME (**pure**) and ME/Ag foams obtained after different reaction times (from 1 to 7 days) using 0.85 mg/mL as the precursor solution.

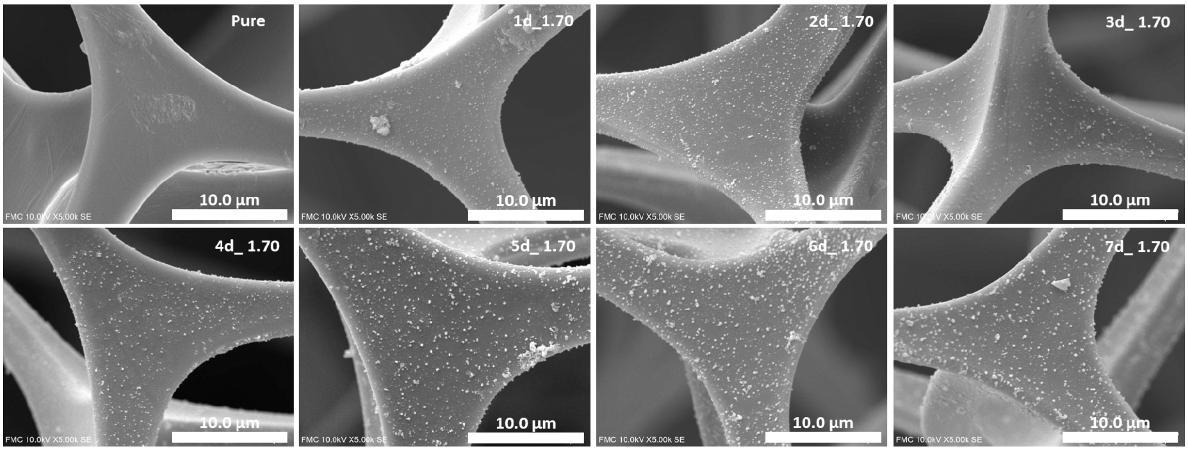

Figure S3. SEM micrographs of the untreated ME (**Pure**) and ME/Ag foams obtained after different reaction times (from 1 to 7 days) using 1.70 mg/mL as the precursor solution.

Figure S4 shows Atomic Force Microscopy (AFM) analysis of pure ME and ME/Ag using 0.85 mg/mL as the precursor solution and 7 day of reaction time. AFM was performed in tapping mode using a Cypher ES AFM from Asylum Research. The data were acquired using a standard AC160TS cantilever from Olympus with a spring constant k of approximately 26 N m^{-1}, a Q factor of 300, and a resonant frequency of 260 kHz. These micrographs show that the ME/Ag foams present rough surfaces (Figure S3b) instead of the smooth surfaces of the ME foams (Figure S3a). Moreover, it is possible to identify that the AgNPs aggregates that can be observed both in SEM micrographs (Figure 1) and low magnification AFM micrographs (Figure S3b) are composed by smaller particles (Figure S3b).

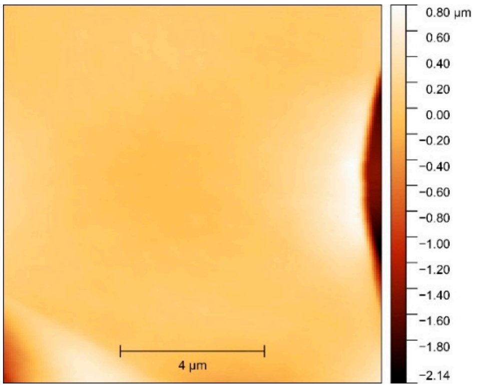

(a)

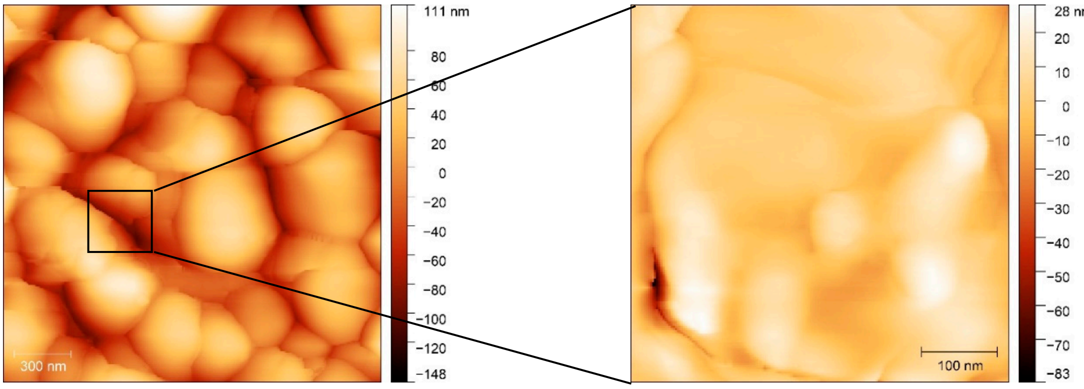

(b)

Figure S4. AFM imagens of ME (**Pure**) (**a**) and ME/Ag foams using 0.85 mg/mL as precursor solution obtained 7 days of reaction times (**b**).

S3. Colorimetry Analysis of ME and ME/Ag Foams

Table S1 summarizes the RGB values for ME and ME/Ag foams obtained using 0.85 and 1.70 mg/mL as precursor solution for different reaction times (from 1 to 7 days).

Table S1. Colorimetry RGB coordinates obtained for the pure ME foam and ME/Ag foams obtained using 0.85 and 1.70 mg/mL precursor solutions as well as different reaction times from 1 to 7 days.

Samples	Reaction time (days)	Precursor solution (mg/mL)	R	G	B
ME_Pure	--		178.77 ± 2.26	184.97 ± 1.82	191.13 ± 2.72
ME_1d-0.85 mg/mL	1	0.85	143.40 ± 4.36	118.35 ± 5.32	99.45 ± 9.16
ME_2d-0.85 mg/mL	2	0.85	131.00 ± 6.19	106.48 ± 5.49	89.60 ± 10.46
ME_3d-0.85 mg/mL	3	0.85	108.65 ± 3.84	83.97 ± 1.58	66.90 ± 1.66
ME_4d-0.85 mg/mL	4	0.85	103.33 ± 1.17	78.38 ± 0.33	63.23 ± 1.07
ME_5d-0.85 mg/mL	5	0.85	96.67 ± 2.20	71.77 ± 2.03	58.94 ± 3.46
ME_6d-0.85 mg/mL	6	0.85	88.90 ± 4.14	63.16 ± 3.98	56.82 ± 6.11
ME_7d-0.85 mg/mL	7	0.85	84.05 ± 1.95	63.45 ± 0.15	53.75 ± 0.45
ME_1d-1.70 mg/mL	1	1.70	138.49 ± 1.17	108.59 ± 2.29	85.92 ± 3.81
ME_2d-1.70 mg/mL	2	1.70	122.92 ± 1.80	92.44 ± 1.09	69.50 ± 0.87
ME_3d-1.70 mg/mL	3	1.70	112.47 ± 2.81	84.17 ± 1.29	63.40 ± 0.70
ME_4d-1.70 mg/mL	4	1.70	104.68 ± 1.61	75.85 ± 0.03	57.90 ± 0.36
ME_5d-1.70 mg/mL	5	1.70	92.72 ± 0.79	67.17 ± 0.51	54.86 ± 0.12
ME_6d-1.70 mg/mL	6	1.70	83.49 ± 1.43	62.06 ± 2.73	52.15 ± 2.40
ME_7d-1.70 mg/mL	7	1.70	81.80 ± 2.19	60.67 ± 0.09	52.80 ± 1.71

S4. Summary of the Obtained Results

Tables S2 and S3 summarize all the AgNPs contents of the ME/Ag foams obtained by the different characterization techniques employed.

Table S2. AgNPs contents determined by ICP-OES of the ME/Ag foams obtained using 0.85 and 1.70 mg/mL precursor solutions as well as different reaction times of 1 3, 5, and 7 days.

Days	AgNPs content (wt.%)	
	ICP-0.85 mg/mL	ICP-1.70 mg/mL
1	1.24±0.10	2.81±0.37
3	4.08±0.78	4.98±0.85
5	8.88±0.60	11.22±0.40
7	13.61±0.32	18.60±0.23

TableS 3. AgNPs contents determined by TGA, Colorimetry, and X-ray radiography of the ME/Ag foams obtained using 0.85 and 1.70 mg/mL precursor solutions as well as different reaction times from 1 to 7 days.

Days	AgNPs content (wt.%)							
	TGA 0.85 mg/mL	TGA 1.70 mg/mL	ΔL 0.85 mg/mL	ΔL 1.70 mg/mL	ΔE 0.85 mg/mL	ΔE 1.70 mg/mL	X-ray 0.85 mg/mL	X-ray 1.70 mg/mL
1	3.59 ± 0.25	2.56 ± 0.34	1.44 ± 0.19	2.03 ± 0.01	1.24 ± 0.39	2.22 ± 0.27	2.05 ± 0.04	1.51 ± 0.28
2	3.50 ± 0.33	4.63 ± 0.41	2.27 ± 0.34	3.74 ± 0.11	1.87 ± 0.89	4.58 ± 0.19	2.94 ± 0.13	1.65 ± 0.17
3	3.18 ± 1.40	3.91 ± 0.97	5.43 ± 0.07	5.20 ± 0.03	5.35 ± 0.73	6.18 ± 0.43	4.89 ± 0.06	5.45± 0.30
4	3.88 ± 0.57	8.09 ± 1.17	6.74 ± 0.32	7.25 ± 0.43	6.68 ± 0.46	8.36 ± 0.71	6.71 ± 0.08	7.26 ± 0.17
5	5.70 ± 0.03	9.81 ± 1.52	8.76 ± 0.12	10.58 ± 0.57	8.76 ± 1.52	10.85 ±0.95	8.22 ± 0.09	9.84 ± 0.99
6	11.42 ± 1.63	14.69 ± 0.54	11.68 ± 1.06	13.64 ± 0.30	11.39 ± 3.86	13.23 ± 1.36	12.45 ± 0.23	14.90 ± 0.48
7	12.44 ± 0.54	18.42 ± 0.41	12.87 ± 0.55	18.20 ± 0.45	12.30 ± 1.09	16.55 ± 0.60	15.31 ± 0.27	17.57 ± 0.40
